# Autophagy in the Immunosuppressive Perivascular Microenvironment of Glioblastoma

**DOI:** 10.3390/cancers12010102

**Published:** 2019-12-31

**Authors:** Maria L. Molina, David García-Bernal, Salvador Martinez, Rut Valdor

**Affiliations:** 1 Brain Regionalization and Development Gene Unit at Biomedical Research Institute of Murcia (IMIB-Arrixaca), 30120 Murcia, Spain; 2 Instituto de Neurociencias UMH-CSIC, CIBERSAM of ISCIII, 03550 San Juan de Alicante, Spain; 3 Internal Medicine Department at University of Murcia-Biomedical Research Institute of Murcia (IMIB-Arrixaca), 30120 Murcia, Spain

**Keywords:** autophagy, chaperone-mediated autophagy, tumor, glioblastoma, perivascular cells, pericytes, immunosuppressive, tumor immune tolerance, autophagy inhibitors

## Abstract

Glioblastoma (GB) has been shown to up-regulate autophagy with anti- or pro-oncogenic effects. Recently, our group has shown how GB cells aberrantly up-regulate chaperone-mediated autophagy (CMA) in pericytes of peritumoral areas to modulate their immune function through cell-cell interaction and in the tumor’s own benefit. Thus, to understand GB progression, the effect that GB cells could have on autophagy of immune cells that surround the tumor needs to be deeply explored. In this review, we summarize all the latest evidence of several molecular and cellular immunosuppressive mechanisms in the perivascular tumor microenvironment. This immunosuppression has been reported to facilitate GB progression and may be differently modulated by several types of autophagy as a critical point to be considered for therapeutic interventions.

## 1. Introduction

Autophagy is essential to maintain cell homeostasis, acting as a protein/organelle quality control mechanism in response to cellular or environmental stresses such as starvation, hypoxia, and chemo/radiotherapy, which is required for cancer-cell survival and the adaptation to the disturbed tumor microenvironment. Autophagy in cancer plays a dual role as a pro-tumorigenic or tumor-suppressive mechanism, which depends on the stimulus and the cell type. The effects of autophagy change in relation to the stage of tumor development and exhibit intratumoral heterogeneity, interfering with the clinical application of autophagy activators or inhibitors. During the initiation of tumor development, autophagy eliminates damaged organelles and aggregated proteins, and prevents DNA damage, protecting cells from transformation into malignant cells and promoting tumor suppression via cellular senescence [1,2,3]. However, once tumorigenesis is initiated, autophagy enhances establishment and progression of the tumor [1,4,5,6,7].

Glioblastoma (GB), the most aggressive brain cancer, evades the immune system [8,9,10,11]. GB cells interact with cells from perivascular areas and invade the brain parenchyma forming a functional network of microtubes [12,13,14,15]. Several mechanisms have been described to favor GB progression. That is the case of stablished interactions between GB cells and tumor surrounding cells, which are essential to communicate and deliver organelles and nutrients, securing tumor survival [13,14,16]. Importantly, the brain immune defense fails during GB progression, preventing anti-tumor immune responses that may be regulated by autophagy. Autophagy keeps the homeostasis of immune cells and modulates the response to stressful stimuli. Autophagy regulates specific immune functions such as antigen presentation, cell activation and differentiation [17,18,19,20]. Thus, as it occurs with anti-cancer direct therapies, the use of autophagy modulators may affect the effectiveness of the current immunotherapies, and could strengthen the immune system to eliminate the cancer cells [21,22,23].

In this review, we will focus on the current findings that identify different forms of autophagy in GB cells and their functions in the tumor progression, with particular attention to perivascular brain areas, where the tumor is established and progresses, escaping from the immune system. We will describe how is the regulation and function of those autophagic pathways in immune cells, which should contribute to the brain defense against GB, but instead, support tumor growth and fail to promote anti-tumor immune responses.

Macroautophagy (also referred to as autophagy), microautophagy and chaperone-mediated autophagy (CMA) are the main known types of autophagy. Those forms of autophagy differ in how are regulated, the nature of the degraded substrates and in how the cargo is targeted to lysosomes. However, there is little data on the functions of endosomal microautophagy in tumor cells [24,25] or in the immune cells that could participate in the response to GB, macroautophagy and CMA are profusely reported to have important roles in those cell types. In this manuscript, we will review these two types of autophagy, focusing especially on CMA as an autophagy pathway that selectively degrades specific proteins that modulate tumor progression in the immunosuppressive perivascular microenvironment, which may constitute a promising target mechanism to treat this aggressive cancer.

## 2. Immune Suppressive Mechanisms in the Tumor Microenvironment

The immune system is highly specialized in the recognition of foreign antigens and unhealthy cells, including tumor cells. However, cancers have developed different strategies to escape immune system recognition and suppress the anti-tumor immune response. Specifically, it is well known that the brain tumor microenvironment is characterized by secretion of a variety of anti-inflammatory molecules, not only by tumor cells themselves, but also by other surrounding peritumoral cells previously conditioned by the tumor. These peritumoral cells are peripheral immune cells (i.e., tumor-associated macrophages (TAMs), T cells, myeloid-derived suppressor cells (MDSC) and T regulatory cells (Tregs)), and various specialized organ-resident cells including microglial cells, astrocytes and perivascular pericytes (PC) [8,10,13]. Collectively, anti-tumor innate and adaptive immune responses in the tumor microenvironment are altered, and several mechanisms including decreased T cell activation and proliferation, induction of anergic T cells, differentiation of Tregs, down-regulation of major histocompatibility complex (MHC) expression and polarization of macrophages to an immunosuppressive M2 phenotype, are prompted by tumor cells [22,26].

The great majority of immune cells infiltrating GB tumor mass are tissue-resident microglial cells and bone marrow-derived macrophages (BMDMs) from circulating monocytes that migrate from blood to brain parenchyma and differentiate to macrophages [22,27]. Together these cells have been called TAM/Ms”. In GB, TAM/Ms displayed a pro-tumorigenic function, producing low amounts of pro-inflammatory cytokines/growth factors and down-regulating expression of co-stimulatory molecules such as CD40, CD80 and CD86 [27]. Moreover, glioblastoma and microglia interactions favor GB progression by secretion of growth factors such as epidermal growth factor (EGF) and colony stimulating factor-1 (CSF-1), or by up-regulation of CD163 and CD204 in infiltrating TAM/Ms, mechanism that has been associated with a worse prognosis. TAM/Ms have been also described to be involved in brain tumor angiogenesis and resistance to anti-angiogenic therapies [28], and to stimulate glioma stem cell invasion through transforming growth factor β (TGFβ) signaling [29]. Other anti-inflammatory cytokine, IL-10, which is related to impaired T cell responsiveness in gliomas, is selectively up-regulated in those tumors with invasive behavior compared to less malignant counterparts [30].

GB cells are also able to avoid anti-tumor T cell responses by several mechanisms, such as prevention of antigen processing and presentation on MHC-class I, up-regulation of PD-L1 expression, the ligand of the negative regulator receptor of T cell activation PD-1 (programmed cell death protein 1), or by increasing infiltration of Tregs and T cells with high expression of PD-1 and CTLA-4 (cytotoxic T-lymphocyte-associated protein 4) [22,31,32].

PC have recently gained special attention due to their role in GB progression. PC are located on the abluminal vessel wall, regulating vascular tone and morphology, and represent an immunological defense in the brain modulating neuroinflammation [33,34,35]. However, PC also express PD-L1 and acquire an immunosuppressive phenotype upon GB interaction, assisting GB to escape the immune system recognition [13,36].

Many studies have provided compelling evidence that autophagy is involved in central nervous system (CNS) tumor recurrence, and in chemotherapy and radiotherapy resistance [37], suggesting that autophagy is other highly regulated mechanism that may contribute to tumor-induced immune tolerance.

## 3. Autophagy in GB

### 3.1. Macroautophagy

**Figure 1:**
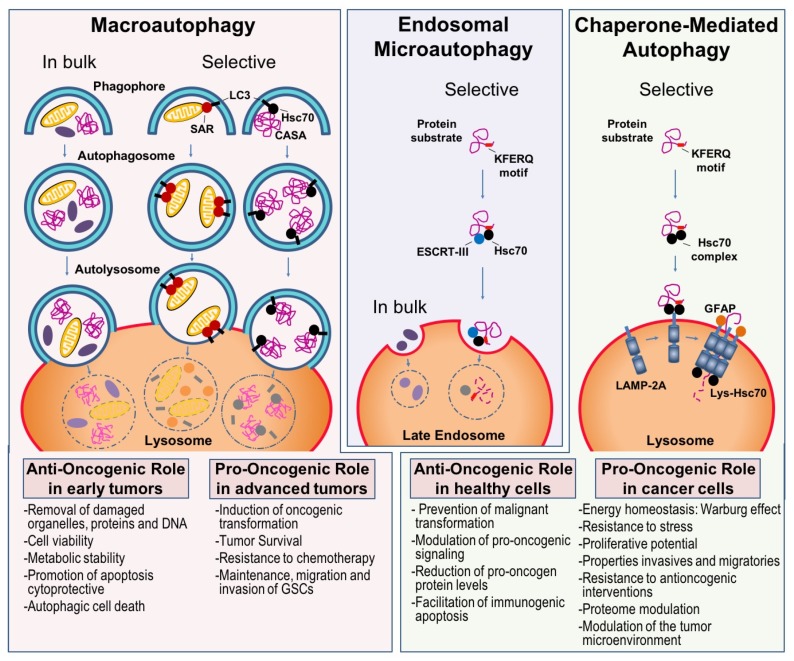
Types of autophagy in mammals and their anti- and pro-oncogenic roles. Macroautophagy, is a process of lysosomal degradation and recycling of cellular components, including damaged organelles, intracellular bacterial pathogens, and aggregated proteins. Macroautophagy sequesters cytosolic contents in de novo-generated double membrane vesicles called autophagosomes that finally fuse with lysosomes. Macroautophagy can be either in bulk or selective for a kind of cargo, and selectivity is mediated by selective autophagy receptors (SAR), or Hsc70 chaperone for protein aggregates in the case of Chaperone-Assisted Selective Autophagy (CASA). Selective macroautophagy binds cargo to the phagophore through lipidated LC3 proteins anchored to the latter’s membrane. Endosomal microautophagy, termed microautophagy in mammalian cells, caters on the degradation of cytosolic regions by late endosomes recognizing the substrates to be degraded, both by bulk degradation of proteins present in cytosol trapped in vesicles forming at the late endosome membrane, and by a selective degradation after binding to Hsc70 chaperone though KFERQ-like motifs. Latter are sorted into intraluminal vesicles in a manner dependent on the endosomal sorting complex required for transport III (ESCRT-III). CMA is a selective process of degradation of soluble, amenable to unfolding, cytosolic proteins presenting KFERQ-like motifs. The chaperone Hsc70 recognizes the motifs and transports the protein substrates to the lysosome where they bind to LAMP2A. LAMP2A multimerizes being stabilized by a glial fibrillary acidic protein (GFAP) and upon unfolding, the substrate is translocated through the lysosomal membrane with the assistance of a lysosomal resident Hsc70, and degraded in the lysosomal lumen. Both macroautophagy and CMA have anti- and pro-oncogenic roles, which depend on the cellular context and are summarized here.

Macroautophagy, also known as autophagy, is a process of lysosomal degradation and recycling of cellular components by sequestering of cytosolic regions in de novo-generated double membrane vesicles known as autophagosomes. Formed autophagosomes end up fusing with lysosomes to degrade their contents. Macroautophagy can be either in bulk or selective. The selectivity is mediated by autophagy receptors that bring cargo to the phagophore, including damaged organelles, intracellular bacterial pathogens and aggregated proteins (Figure 1) [1,2,24,38,39,40,41].

#### 3.1.1. Regulation

Under normal conditions, basal level of autophagy is very low. Therefore, autophagy must be induced through an efficient mechanism to survive stress and adapt extracellular signals. In mammalian cells under nutrient-rich conditions, autophagy is inhibited by the serine/threonine-protein kinase mTOR (mammalian target of rapamycin). mTOR negatively regulates other serine/threonine kinases, Unc-51-like kinase-1 (ULK1) and -2 (ULK2), phosphorylating and inactivating Unc-51-like kinases (ULKs) and autophagy-related gene 13 (Atg13) by binding to the ULKs-Atg13-FIP200 complex [42].

Under conditions of nutrient stress, AMP-dependent protein kinase (AMPK) increases autophagy by inhibition of mTOR complex 1 (mTORC1), via phosphorylation of its Raptor subunit. Then, ULK1 and ULK2 are activated and phosphorylate Atg13 and FIP200. The beclin1 complex is recruited and activates the class III PI3K VPS34, stimulating the autophagosome nucleation [42,43]. In addition, other studies demonstrate that inhibition of mTORC1 promotes the transcription factors EB (TFEB) and E3 (TFE3) nuclear translocation, inducing expression of autophagy–lysosome-relevant genes [44,45]. Autophagy is also induced by Exchange protein directly activated by cAMP 1 (Epac-1) through a Ca^2+/^calmodulin-dependent kinase kinase b (CaMKKb)/AMPK signaling pathway [46]. Recently, inositol polyphosphate multikinase (IPMK) has been reported to induce autophagy through the regulation of AMPK/ULK activation and by enhancing autophagy-related transcription dependent of Sirt-1 activation [47].

In tumor cells, there are many signaling pathways that participate in the regulation of autophagy. Down-regulation of STAT/BCL2/BECLIN-1 [48,49,50] and PI3K/Akt/mTOR-mediated signaling pathways can activate macroautophagy [51,52,53]. The Ras/Raf/ERK signaling pathway is one of the most frequent pathways that activate macroautophagy in tumors [54,55]. Other important modulators of macroauthophagy in GB are Sirt1 that induces autophagic cell death and mitophagy [56], insulin, p53 [57], p38 mitogen-activated protein kinase (p38-MAPK) [58], 5′ AMPK [59], phosphatase and tensin homolog deleted from chromosome 10 (PTEN) [60], and reactive oxygen species (ROS)-associated pathways [61]. Furthermore, intracellular calcium signaling seems also an important regulatory pathway in GB [62,63]. Recent advances in Hedgehog, NRF2-P62, and PD-L1/PD1 signaling pathways seem to indicate that these might be potential targets to modulate macroautophagy in glioma [64,65,66].

It is important to highlight that in cancer cells, in general, acute amino acid starvation induces two responses in parallel: mTOR inactivation with subsequent activation of in-bulk macroautophagy, and induction of an immediate selective endosomal microautophagy independent of mTORC1 inactivation and canonical macroautophagy [24] (Figure 1). Both autophagic pathways likely work in concert and may partially compensate for each other [67].

#### 3.1.2. Functions

Most cells sustain low basal levels of macroautophagy that serve as a quality control system to selectively degrade labeled damaged cargo [41,68,69,70,71,72]. However, autophagy plays a dual role in GB as in other types of cancer, mediating oncogenic or oncosuppressive effects, acting as a “double-edged sword” in tumors, depending on the cellular context and tumor type, the stage of tumor, cell viability and intracellular environment [1,2,3,6].

In the early stages of oncogenic transformation, damaged proteins and organelles are efficiently removed by macroautophagy, maintaining cell homeostasis, metabolism, and viability, which prevents further tumor development [2,3]. Macroautophagy also prevents the accumulation of toxic molecules, such as ROS produced by dysfunctional mitochondria, in addition to aggregates of ubiquitinated proteins [73]. In addition to cytoprotective effects, autophagy exerts a death stimulation function known as autophagy-dependent cell death, which is due to an excessive degradation of cell components and organelles [3,74]. Moreover, several studies indicate that macroautophagy is activated in response to several stressors, promoting glioma apoptosis [73,75,76].

Regarding the role of macroautophagy in epithelial-mesenchymal transition (EMT) of GB stem cells, several studies implicate it in the promotion of EMT, while other works controversially suggest that its inhibition may promote EMT and induce cancer-cell invasion [73,77,78].

During the advanced stages of tumors, macroautophagy is up-regulated, playing a crucial role in the survival and development of GB, providing metabolic support and preventing senescence [6,7,79,80,81], conferring resistance to chemotherapy, as well as supporting the maintenance, migration and invasion of GSCs [73,82,83].

Therefore, treatment of tumors via macroautophagy regulation is extremely complicated because it can be involved in cancer-cell survival or death. In addition, controversial results on macroautophagy quantification in GB still exist in the literature. In fact, misinterpretations often occur when assessing macroautophagy activity, which is often quantified by an increase in LC3 levels, which does not necessarily reflect increased macroautophagy, since it may be due to an accumulation of autophagosomes that do not fuse with lysosomes. Thus, the assessment of macroautophagy status depends on a careful evaluation of several macroautophagy markers, such as LC3-I/II ratio, subcellular localization, Atg12-Atg15 accumulation, and p62 degradation along with an assessment of the macroautophagy flux [84].

### 3.2. Chaperone-Mediated Autophagy

CMA is a selective process of lysosomal degradation of soluble cytosolic proteins with KFERQ-like motifs. The chaperone heat shock cognate 71 kDa protein (Hsc70) binds to the substrate protein by recognition of the specific motifs and carry it to the lysosome with the help of another cochaperones. The protein binds to the CMA receptor at the lysosomal membrane, the limiting component of this pathway known as lysosome-associated membrane protein type 2A (LAMP2A). The protein is unfolded and translocated into the lysosome to be degraded, also contributing other resident Hsc70 in the lysosomal lumen (Figure 1) [85].

#### 3.2.1. Regulation

Basal CMA levels are detectable in almost all types of mammalian cells, and its regulation is critical to maintain cellular function and homeostasis. CMA failure leads, instead, to intracellular accumulation of damaged proteins, defective regulation of many cellular functions, and failed responses to different stressors [86,87,88,89]. CMA in tumor cells seems to be constitutively up-regulated [5]. Starvation, hypoxia, and ROS, characteristics of the tumor microenvironment, activate CMA, but the mechanisms of increased CMA activity in cancer cells are still poorly understood [87,89,90,91,92,93,94].

Although there are no studies on how CMA is regulated in GB cells, it is interesting to consider that mechanisms of CMA up-regulation in some cancer cells are the same as in non-transformed cells [95,96]. In fact, during starvation, normal cells increase the levels of lysosomal LAMP2A by reducing its lysosomal turnover [97], and in a same way, colorectal cancer cells up-regulate LAMP2A by reducing their degradation through lowering levels of sorting nexin 10 (SNX10) [95]. Conversely, other cancer cells use similar mechanisms as healthy cells, but with different results [98]. That is the case of Hsp90 inhibition, which causes reduction of CMA activity by decreasing LAMP2A stabilization at the lysosomal membrane in non-transformed cells, whereas in pancreatic cancer cells, Hsp90 inhibition induces CMA activity and promotes IGF-1Rβ degradation [98]. In other cancer cells, pathways that down-regulate CMA in normal cells, appear not to be functional. That is the case of several cancer-cell lines that up-regulate CMA even in the presence of TORC2/AKT1 signaling pathway, which has a CMA inhibitory effect in normal cells [99].

In most cell types, CMA blockade in vitro and in vivo causes the activation of macroautophagy and the proteasome system [88,100]. Vice versa, the inhibition of macroautophagy or proteasome activates CMA [101,102,103,104], which could be important for the maintenance of cell quality control and energy balance at baseline conditions. However, although some cancer cells retain this autophagic crosstalk, in most cancer cells, CMA is constitutively activated independently of the status of macroautophagy [5].

#### 3.2.2. Functions

CMA is implicated in a wide variety of cellular functions such as protein quality control, response to starvation, anti-ageing functions, transcriptional regulation, immune response, cell cycle and cancer biology (Figure 1). CMA blockage in cancer cells has anti-cancer activity, decreasing cell proliferation and tumorigenic/metastatic ability [4,5]. However, both reduced and increased CMA activity can be pro-oncogenic depending on the cellular context, as reduced CMA increases DNA damage and decreases proteostasis [94], providing an environment that facilitates malignant transformation [105]. However, once cells have undergone transformation, they up-regulate CMA to protect against oxidative damage [4], degrade negative regulators of cell proliferation [106] and anti-oncogenes [107] and maintain the metabolic switch favorable for cancer-cell growth [108,109]. Abnormally high levels of CMA are shown in many cancer-cell lines and different types and stages of human tumors [4,5,110]. In fact, genetic blockage of CMA in cancer-cell lines reduced their tumorigenic ability and CMA inhibition in preformed xenografted tumors induced tumor shrinkage and lowered metastasis number [5].

Many cancer cells need CMA up-regulation to sustain aerobic glycolysis (Warburg effect), required for cancer progression [5,109]. CMA plays an important role under hypoxia conditions in the regulation of the tumor metabolism by degrading cell cycle regulatory proteins such as phosphorylated serine/threonine-protein kinase (CHK1) [111] and hypoxia-inducible factor 1α (HIF1α) [112,113]. In addition, quantitative proteomics analysis of isolated lysosomes from CMA-activated conditions in cancer cells has recently revealed a new role for CMA in the control of cell translation [114]. Importantly, up-regulated CMA may also assist chemoresistance of GB, making them more resistant to different stressors such as oxidative stress, hypoxia or even DNA damage agents [4,115]. Up-regulation of CMA in GB cells that are in contact with perivascular areas, not only favors their proliferation, but it may even interfere with the aberrant CMA up-regulation in tumor surrounding cells that benefits tumor survival and affects anti-tumor immune microenvironment responses [36].

## 4. Autophagy in Immunosuppressive Cells during GB Progression

Immune cells can be modulated during tumor progression by autophagy activation or inhibition, allowing them to promote anti-tumor immune responses or to fail in tumor clearance by inducing tumor immune tolerance. Next, we describe autophagy in the different types of cells best known to contribute in the immunosuppressive microenvironment of GB.

### 4.1. Macroautophagy in Innate Immune Response

**Figure 2:**
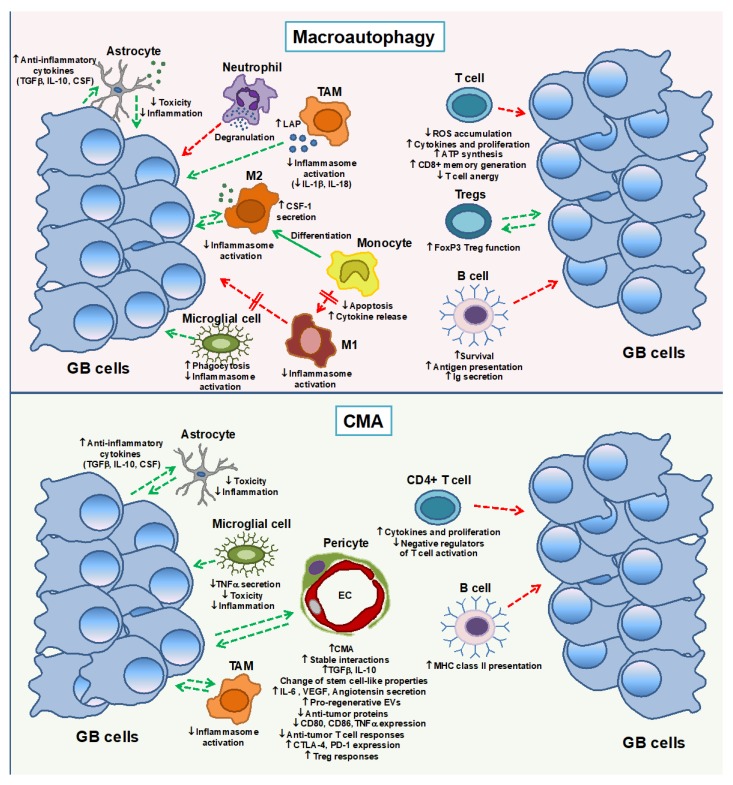
Autophagy function in the immune responses of peritumoral cells during GB progression. Macroautophagy and CMA activation in different immune or brain-resident cells, represents a key point of regulation to favors progression of tumor cells (green arrows) or to promotes its anti-tumor activity (red arrows), respectively. Macroautophagy and CMA up-regulation support tumor progression by increasing phagocytosis and by inhibiting inflammasome-mediated responses of TAMs and microglial cells, and by stimulating differentiation of monocytes into anti-inflammatory M2 macrophages. However, macroautophagy promotion hinders polarization of monocyte into pro-inflammatory M1 macrophages, which may represent an indirect mechanism to advantage tumor progression. Astrocytes have direct physical contact with tumor cells whereas macroautophagy/CMA activity in this cell type contributes to its anti-inflammatory phenotype. Neutrophils require macroautophagy to exert its anti-tumor activity. Regarding the adaptive immune responses, T cells has been shown to require macroautophagy and CMA to develop its anti-tumor activity by regulation of several immune checkpoints (i.e., increasing cytokine release, proliferation, energy store mobilization, and degradation of negative regulators of T cell activation or by prevention of T cell anergy). Macroautophagy and CMA are also necessary for maintaining B cell-specific functions such as antigen presentation. However, macroautophagy promotion favors tumor tolerance by stimulation of FoxP3 T regulatory cell function. GB-induced CMA modulates pericytes immune function through cell-cell stable interactions promoting GB survival and progression. GB-conditioned pericytes display an aberrant up-regulation of CMA that lead to secretion of anti-inflammatory cytokines, angiogenic molecules, pro-regenerative extracellular vesicles, and prevention of anti-tumor proteins secretion that benefits tumor growth. Furthermore, GB-induced CMA in PC down-regulates expression of co-stimulatory molecules, prevents pro-inflammatory cytokine secretion and fails to promote anti-tumor T cell responses, enhancing Treg responses, which contributes to the immunosuppressive peritumoral niche of GB. Ig: immunoglobulins; EVs: extracellular vesicles; EC: endothelial cells.

Macrophages and microglia are the main innate immune cells of the CNS which usually facilitate tumor-mediated immunosuppression and tumor progression [22,27]. Infiltrated macrophage populations contribute to up to 50% of non-neoplastic cells in the tumor microenvironment [27] and require autophagy to maintain immune homeostasis. Macrophages use LC3-associated phagocytosis (LAP), a type of non-canonical autophagy, to eliminate cells captured in phagosomes that fuse to the lysosome, during tissue maintenance [18]. The failure of this pathway promotes impaired degradation of phagocytosed cells, inducing systemic inflammatory disease that can help anti-tumor immunity [17] (Figure 2). Induction of autophagy by activating c-Jun N-terminal kinase (JNK) and blocking ATG5 cleavage is essential for differentiation of monocytes into macrophages, preventing monocyte apoptosis and inducing cytokine production in response to granulocyte-macrophage colony-stimulating factor (GM-CSF) [116]. Autophagy also plays an important role in MDSC polarization. The inhibition of autophagy in macrophages (i.e., by knocking out *Atg5* gene expression) promotes M1 polarization [117,118]. Furthermore, activation of autophagy in response to colony-stimulating factor-1 (CSF-1) and through AMPK signaling causes monocytes to differentiate into immunosuppressive M2-like macrophages, contributing to tumor progression [17]. Blocking autophagy due to decreased ATG16L1 expression enhances production of the pro-inflammatory cytokines IL-1β and IL-18, suggesting that autophagy regulates inflammasome activation and controls production of those cytokines [119] (Figure 2).

By contrast, neutrophils, other type of myeloid-derived cells that can develop an immunosuppressive function in GB [120], require macroautophagy to induce inflammation [121,122]. Microglia, the tissue-resident macrophage population of the brain, also require autophagy to maintain their ability to phagocytose apoptotic cells, protein aggregates and debris, and its failure enhances inflammation as it occurs in macrophages [17]. Several publications show activation of primary mouse microglia or microglial cell lines after knockdown of autophagy genes (i.e., *Atg5* or *Atg7*) [123,124,125], and characterize how the inflammatory response can be modulated by Beclin1-driven autophagy through NLRP3 degradation [126] (Figure 2).

Reactive astrocytes establish direct cell interactions by gap junctions with tumor cells and microglial cells at the peritumoral glial scar favoring tumor progression and chemoresistance. Astrocytes contacting tumor cells secrete cytokines that support tumor metastasis in the brain and contribute to an immunosuppressive microenvironment with high levels of anti-inflammatory cytokines such as IL-10, TGFβ and CSF [10]. Autophagy/lysosomal dysfunction in astrocytes participates in neurodegeneration as observed in lysosomal storage disorders [127]. However, little is known on the possible role of macroautophagy in the immune function of astrocytes. Recent works show that this process is needed for the homeostasis maintenance upon stimulation, but depending on the type of stressor, it can also facilitate cell death and therefore brain inflammation [128,129,130] (Figure 2).

In summary, inhibition of macroautophagy in MDSC and glia lead to a pro-inflammatory response that might alert the immune system to regress the tumor progression. However, it is important to consider that macroautophagy inhibition can also impair phagocytosis and promote high toxicity and inflammation, which can contribute to neurodegeneration associated with different diseases [125,126].

### 4.2. Macroautophagy in the Adaptive Immune Response

T cells, which can infiltrate the brain to promote tumor clearance, are found in peritumoral areas during GB progression, being affected by several immune checkpoints that impair effective anti-tumor responses. Macroautophagy role is essential in the maintenance of T cell homeostasis for the turnover of organelles such as mitochondria. Macroautophagy prevents increased ROS accumulation that lead to higher rates of cell death [131,132]. Macroautophagy inhibition deleting *Atg* gene or using chemical inhibitors negatively impacts the responses to antigen. Thus, it impairs activation-induced proliferation upon T-cell receptor (TCR) engagement, which is associated with fast increased calcium levels [133]. Furthermore, recent works have shown selective degradation of inhibitors of cyclin-dependent kinases or TCR signaling proteins, which contribute to T cell proliferation [132,134]. Importantly for tumor progression, the accumulation of the protein tyrosine phosphatase PTPN1 in autophagy-deficient CD4^+^ T cells produces failed T cell responses upon priming and also after subsequent stimulation, which seem to indicate that macroautophagy also regulates T cell tolerance [134]. Interestingly, IL-2 receptor signaling enhances macroautophagy in peripheral CD4^+^ T cells by increasing LC3 expression, whereas IFN-γ, T helper 1 cells signature cytokines, promotes macroautophagy in macrophages via the p38 MAPK signature pathway [135,136] (Figure 2).

Autophagy maintains the energy demands of the metabolism of CD4^+^ T cells, contributing to sustain adenosine triphosphate (ATP) production in response to TCR engagement, proper anaerobic glycolysis and mitochondrial respiration [133,134]. Autophagy-related (ATG) proteins-dependent autophagic pathways also modulates T cell differentiation and function, regulating the generation of different T cell populations [20]. Autophagy is also needed in FOXP3^+^ regulatory T cells (Treg) to suppress anti-tumor immune responses, maintaining Treg cell homeostasis by prevention of metabolic alterations that decrease their survival and may lead to autoimmunity [137]. Importantly, CD8^+^ T cell memory generation and maintenance require of autophagy activity [138]. Recent works indicate that the ability of autophagy to reprogram CD8^+^ T cell metabolism, contributes in modulation of the efficacy of anti-tumor CD8^+^ T cell responses [139,140] (Figure 2).

Less has been reported about B cells in GB; however, it is important highlight that this type of cell may possibly infiltrate GB during progression or regression after therapy, since they can act as antigen-presenting cells (APCs) and may modulate tumor antigen-specific T cells [141,142]. IL-4, a signature cytokine of T helper 2 cells can induce autophagy in B cells via a PI3K-dependent pathway, promoting survival and antigen presentation of B cells [143]. Furthermore, macroautophagic activity regulates B cell development, and its failure, leads to defective humoral immune response with reduced secretion of immunoglobulins and failed activation upon the engagement of the B cell receptor (BCR) [144,145] (Figure 2).

### 4.3. CMA in Innate Immune Response

Although little is known today about the role of CMA in the innate immune response, and even less in the GB microenvironment, recent studies point to an increase in CMA in astrocytes and microglia, as a protective mechanism against pathogenic crosstalk with neurons that causes toxicity, inflammation and neurodegeneration [146,147]. A recent study in TAMs shows that LAMP2A expression is up-regulated by tumor cells in several mouse tumor models and cancer patients’ samples. Suppression of *Lamp2a* gene expression by either shRNA or CRISPR/Cas9 prevents TAMs activation and tumor growth, whereas substrates degradation by CMA seems to promote macrophage pro-tumorigenic activation [148] (Figure 2).

Recent studies of our group have revealed a new role of CMA in the immune function of perivascular cells PC during GB progression and through interaction with tumor cells [13,36]. PC initiate aberrant degradation of proteins in response to a burst of oxidative stress upon GB interaction, changing the levels of secretion of several cytokines and of growth factor release, in a manner that supports tumor cell proliferation and GB survival. GB induces CMA in PC to modulate stem-cell-like properties and prevent secretion of anti-tumor proteins, facilitating tumor growth. Furthermore, GB-induced CMA in PC enhances secretion of the anti-inflammatory cytokines TGFβ and IL-10 to promote an immunosuppressive phenotype that leads to failed tumor antigen-specific T cell responses, facilitating tumor tolerance. In a mouse model of xenografted GB cells, PC with impaired CMA, by knocking out *Lamp2a* expression, promote antigen-specific T cell infiltration in the peritumoral areas, resulting into tumor elimination. FOXP3^+^ Tregs populations hardly were infiltrated in those areas and reduced expression of immune checkpoints, such as CTLA-4 and PD-1 in T cells, was detected [36]. This work supports that CMA inhibition in specific cells of the innate immune response of the brain, such as PC, may be an effective approach to treat this aggressive disease (Figure 2).

### 4.4. CMA in the Adaptive Immune Response

So far, the role of CMA in CD8^+^ T cells in anti-tumor immunity is unknown. In CD4^+^ T cells, CMA is essential for T cell activation, and its failure has been shown to impair T helper cell responses to immunization and to infection by pathogens such as *Listeria monocytogenes*. CMA selectively degrades two negative regulators of T-cell activation, the itchy homolog E3 ubiquitin protein ligase (ITCH) and the regulator of calcineurin 1 (RCAN1), following stimulation of the TCR. High levels of those CMA substrates are accumulated in CMA-deficient T cells and are responsible for decreased cytokine release and T cell proliferation upon T cell activation [89] (Figure 2). Importantly, decreased or dysregulated CMA with age in T cells, which also occurs in macroautophagy, may translate into altered quality control, energy balance and proteome remodeling. Therefore, it would be also important to consider the age to modulate autophagy in future therapies of GB that may affect T cells [20].

Finally, the important role of CMA in MHC-class II presentation of cytoplasmic antigens by B cells [149,150] should be also considered during GB progression [141,142], since CMA in B cells might facilitate anti-tumor T helper responses (Figure 2).

## 5. Are Drugs Targeting Autophagy an Effective Anti- GB Tumor Therapy?

Despite significant advances in conventional first-line therapeutic strategies for primary GB (i.e., surgical resection, radiotherapy and chemotherapy), tumor reappears in almost all patients, showing a median survival of 12-15 months from initial diagnosis. Therefore, new therapeutic approaches enhancing effectiveness of GB treatment are urgently needed.

As mentioned previously, macroautophagy and CMA are up-regulated in most tumors [5,53,94,151,152], being the first even more increased after radiation and chemotherapy [37]. Autophagy has been considered to be a promising therapeutic mechanistic target to prevent progression of a variety of tumors including GB [36,81]. Chloroquine and hydroxychloroquine are the only available macroautophagy inhibitors in the clinical practice and they have already been used in several clinical trials, alone or in combination with temozolomide (TMZ), showing a significant increased survival in patients with GB [153,154]. Other reported macroautophagy inhibitors that sensitize GB cells to chemotherapy are 3-methyladenine, wortmannin, bafilomycin A1 or siRNA for knocking down expression of *Rab7* or *Atg7* genes [155,156,157]. However, it must be taken in consideration that autophagy activation may also have deleterious effects on tumor cells, as a persistent stimulation of autophagy may result in cell death. That is the case of rapamycin, LY294002, arsenic trioxide or tamoxifen [157]. Combination of drugs which increase autophagic flux such as tricyclic antidepressants (TCAs) and certain anticoagulants (inhibitor of the purinergic receptor P2Y12) promotes autophagy-associated cell death in glioma cells [158].

Considering that CMA regulatory mechanisms in some cancer cells act differently than in healthy cells [94] (Figure 1), several CMA control points reported must be validated in each type of cancer: (1) the first one is constituted by three proteins: mTOR2, Akt, and PHLPP1. While mTOR2 and Akt inhibit CMA, dynamic recruitment of PHLPP1 to lysosomal membrane neutralizes this inhibitory effect and activates CMA. This signaling axis may be blocked by using a selective inhibitor of PHLPP1 [99]; (2) under conditions of oxidative stress, the transcription factor known as nuclear factor of activated T-cells (NFAT) induces *Lamp2* expression, an effect that is prevented by the calcineurin inhibitor cyclosporine A or by blockade of ROS production by the anti-oxidant N-acetyl-cysteine [89]; (3) the transcription factor nuclear factor, erythroid derived 2, such as 2 (NFE2L2/NFR2) regulates expression of *Lamp2a*, being the NFE2L2 deficiency associated with reduced LAMP2A levels [159]; (4) retinoic acid receptor alpha (RARα) has been found to negatively regulate LAMP2A expression, and the RARα activator all-trans retinoic acid (ATRA) acts as a potent LAMP2A inhibitor [160]; (5) endoplasmic reticulum (ER) stress mediates p38MAPK activation which in turns phosphorylates LAMP2A and promotes CMA, a mechanism termed “ERICA” (ER stress-induced chaperone-mediated autophagy) [161]; (6) Phosphopeptide P140, a fragment of the spliceosomal SNRNP70/U1-70K protein, has been reported to alter the composition of HSPA8/HSC70 complexes and down-regulates LAMP2A expression, thereby reducing CMA [162]; and (7) Unlike LAMP2A, the only LAMP2 isoform that participates in CMA, LAMP2C negatively regulates melanoma growth and survival [163].

Antibiotics, such as cycloheximide, anisomycin and SB230580 seem to affect CMA activity whereas geldanamycin acts as a CMA activator in some contexts [164], but selectively inhibits chaperone Hsp90 in others, inhibiting CMA in some types of tumor cells and showing anti-cancer activity [165,166]. However, as some of the cell components that control macroautophagy and CMA at different levels are also involved in other signaling pathways that regulate key cellular processes other than autophagy, pharmacological activation, or inhibition of any of these proteins could have significant non-specific unwanted adverse effects.

Conversely, and although specific chemical inhibitors of CMA are not currently available in clinic, expression levels and assembly of LAMP2A to the lysosomal membrane represent the only limiting factor to regulate CMA activity [88]. In this regard, gene therapy directed to CMA only by blocking LAMP2A expression could provide a much greater specificity to this type of anti-cancer strategies. Thus, it has been found that blockade of LAMP2A expression delays growth and inhibits metastasis of a variety of solid tumors [4,5,94,167]. In the case of GB, it has been recently addressed for the first time that GB not only increase its CMA to survive and progress but GB cells also induce an abnormal up-regulation of basal CMA in PC of brain peritumoral areas, to modulate PC immunosuppressive phenotype through cell-to-cell interaction and facilitate GB progression [36]. Accordingly, inhibition of CMA in PC (i.e., by knocking out LAMP2A expression) destabilizes GB-PC interactions and promotes a secretory function on PC that contributes to removal of GB cells, a strategy that may be considered to be a promising new therapy against GB.

In summary, the use of pharmacologic inhibitors or genetic therapy to inhibit macroautophagy may prevent GB progression, but also promotes tumor immune tolerance. Therefore, future therapy in brain peritumoral areas against *Lamp2a* expression could inhibit more specifically GB survival, through impaired CMA, not only in the tumor cell but even in cells that interact with the tumor, preventing the immunosuppressive microenvironment that facilitates tumor survival.

## 6. Conclusions and Future Perspectives

It must be considered that systemic administration of any autophagy inhibitor/activator will not only affect tumor cells, but also all cell types present in the peritumoral microenvironment, a situation that could lead to the occurrence of serious side effects. Moreover, the possible anti-tumor strategy to be developed is further complicated considering that depending on the type of tumor cell or tumor stage, macroautophagy could act as a pro- or anti-tumorigenic mechanism [3,73,81], a situation that hinders the eligibility of an inhibitor or an activator of macroautophagy. CMA could represent a more specific targetable mechanism to prevent GB progression, considering that current macroautophagy inhibitors may have effects on many other cell processes different to macroautophagy, while targeting *Lamp2a* gen may be specific for CMA.

Peritumoral areas, where GB progresses and where we can find tumor interactions with other cells facilitating tumor growth, are promising targets to be considered for new treatments after tumor surgery. As discussed above, genetic manipulations to block CMA in tumor cells or alternatively in peritumoral cells could be a suitable therapeutic option to prevent tumor survival and avoid its progression. However, CMA activity is also needed to maintain effector functions of T cells [89], which are of key importance for complete tumor removal. Collectively, the ideal treatment for GB could be constituted by a strategy that pursues the selective inhibition of CMA in the tumor and in the peritumoral microenvironment, specifically in those cells in which CMA favors tumor progression such as PC. Not less important would be to combine this treatment simultaneously with other therapeutic strategies that boost tumor antigen-specific T cell responses, allowing the complete removal of the tumor. Combined immunotherapies able to cross blood-brain barrier or intrathecal therapy [23] (i.e., monoclonal antibodies, peptides, pro-inflammatory cytokines, dendritic cell vaccines, high dose IL-2 (HDIL-2) immunotherapy or chimeric antigen receptor T (CART) cell therapy against GB [168,169]) could lead to improved therapeutic results.

In conclusion, CMA could be considered to be a critical mechanism to be exploited as a promising new gateway to enable an efficient anti-GB therapy.
